# Quantum Dot Nanomaterials: Preparation, Characterization, Advanced Bio-Imaging and Therapeutic Applications

**DOI:** 10.1007/s10895-023-03472-0

**Published:** 2023-10-25

**Authors:** Marwa Nabil, Fayed Megahed

**Affiliations:** 1 Department of Electronic Materials Researches, Advanced Technology and New Materials Research Institute, City for Scientific, Research and Technology Applications, Alexandria, 21934 Egypt; 2 Nucleic Acid Research Department, Genetic Engineering and Biotechnology Research Institute, City of Scientific Research and Technological Applications, Alexandria, 21934 Egypt

**Keywords:** Quantum dot Techniques, Quantum dot Applications, Bio-imaging, Fluorescence Emission, Vivo Imaging

## Abstract

The bio-imaging technology is one of the most significant modern applications used in several fields, including early diagnosis of many illnesses that are most important diseases facing humanity and other vital uses. The primary advancement in nanotechnology is the creation of innovative fluorescence probes called quantum dots (QDs). The use of molecular tagging in research, in vivo, and in vitro studies is revolutionized by quantum dots. The application of QD indicates conversion in natural imaging and photography has demonstrated extraordinary appropriateness in bio-imaging, the discovery of novel drugs, and delivery of targeted genes, biosensing, photodynamic therapy, and diagnosis. New potential methods of early cancer detection and treatment management are being researched as a result of the special physical and chemical characteristics of QD probes. The bio-imaging technique depends on the fluorescent emission of the used materials, which is paired with living cells that are easy to see it in 3D without any surgical intervention. Therefore, the use of QDs many types that have unique and appropriate properties for use in that application; In terms of fluorescent emission strength, duration and luminosity.This review article displays some methods of preparation for QDs nanomaterials and the devices used in this. In addition, it presentssome of challenges that must be avoided for the possibility of using them in the bio-imaging field; as toxicity, bio-compatibility, and hydrophilization. It’s reviewed some of the devices that use QDs in bio-imaging technique, the QDs application in cell analysis-imaging, and QDs application in vivo imaging.

## Introduction

The quick evolution of fluorescent nanomaterial during the past three decenniums has led to increased biological and medical research into their potential applications [1]. Fluorescence nanomaterialsare a very important sort of the colloidal semiconductor quantum dots (QDs), which has a dimension in size scale in between 5 and 10 nm [2]. By a simple comparison with organic fluorophores, QDs have approximately 20 times brightness and 100 times more photo-stable than standard fluorescent dyes [3]. So, QDs have an important function in the imaging technique and extremely fluorescent probes for biological applications. It has topmost sensitivity, prolonged stability, good biocompatibility, and minimal invasiveness [4]. So, there are two main points for using it as a rostrum for imaging applications; its high photoluminescence quantum yield (PL QY) and its optical properties gauge that can be modified with a bit changing their size [5]. It is often used in these applications, metal elements(i.e. Ni, Co,.) or mostly on the broad scale of semiconductor materials, particularly from group II-VI in periodic table (i.e. CdS, ZnS, CdSe, ZnSe, CdTe, .). But regarding the different semiconductor elements in group III-V (i.e. In, Ga,.), can be used for different QDs applications [6]. But, it makes clinical application difficult as a result of its toxicity [7]. Despite the QDs toxicity can be partially lessened by instilling the toxic core in polymers or silica to form a core-shell structure [8], so encapsulation methods produce in larger hydrophilic QDs (~ 20–30 nm) [9].

**Fig. 1:**
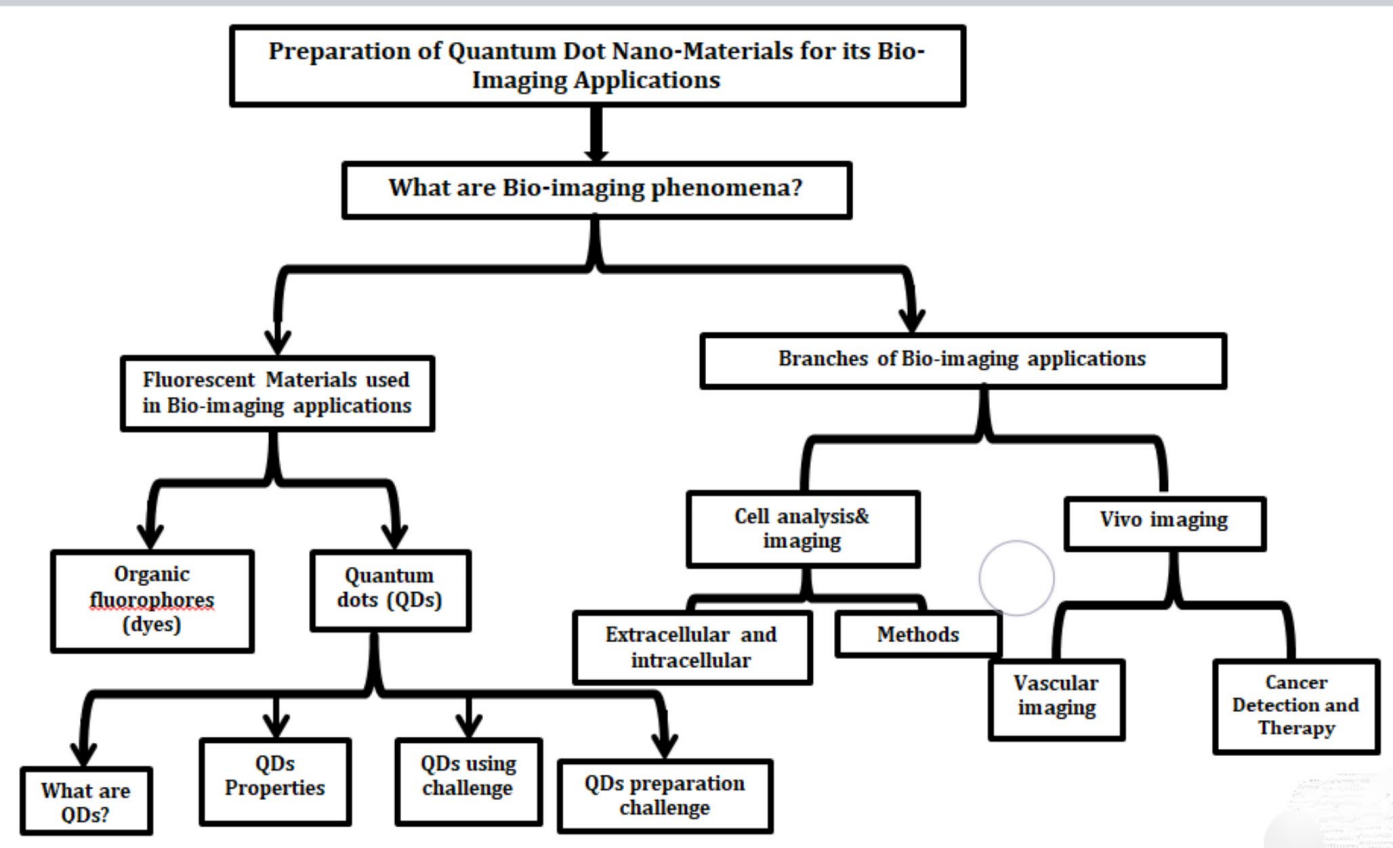
Flowchart of challenge in preparation of quantum dot nanomaterials for his bio-imaging applications

In this article, we first present several previous reviews discussed the bio-imaging applicationsand the most challenge points that face researchers for fabrications of it using quantum dotnanomaterials. In the last section, the order of Egypt and other countries in using QDs nanomaterials in bio-imaging applications are discussed, for characterization in Vitro and Vivo, as shown in Fig. 1.

## Bio-imaging Phenomena

From the 1960s till now, the image treating technology was improved, nowadays; a massive growth rate in the use of image processing technology can be seen. It is the technology with the ability to replace the human vision by an artificial vision for observation of objects and processes. Lately, the treated image of the biological applications is understood by researchers, and many trials are made to focus on conducting research for the employment of this technique in biological imaging.

Biological imaging (Bio-imaging) is defined as an accurate technique for the information enrollments of relevant to biological materials using a set of imaging equipment and processing. Ordinarily, it is defined as a procedure for recording the biological process for the enrollment of the biological specimen information [10].

Thus, imaging is now the controlling form of the analysis of molecules, cells, and tissues across the Life Sciences.***Bio-imaging*** used to gain information on the 3-D structure of the observed specimen from the outside, i.e. without physical interference.It relates to methods that non-invasively visualize biological processes in real-time. And, it aims to interfere as little as possible with life processes. Also, Bio-imaging includes methods of visualizing treated biological material, as subcellular structures and entire cells over tissues in multicellular organisms.In addition, it uses different sources of imaging, I. e. Light, fluorescence, electrons, … [11].

## Materials used in Bio-imaging Applications

At dipping in cells, these cells turn into self-reporting for the metabolite in question [11, 12]. As previously shown, the bio-imaging technique depends on the light emission of the different cell surfaces, materials that have the property of light emission as fluorescent emission.

### Organic Fluorophores Compounds (i.e. Organic dyes)

**Fig. 2:**
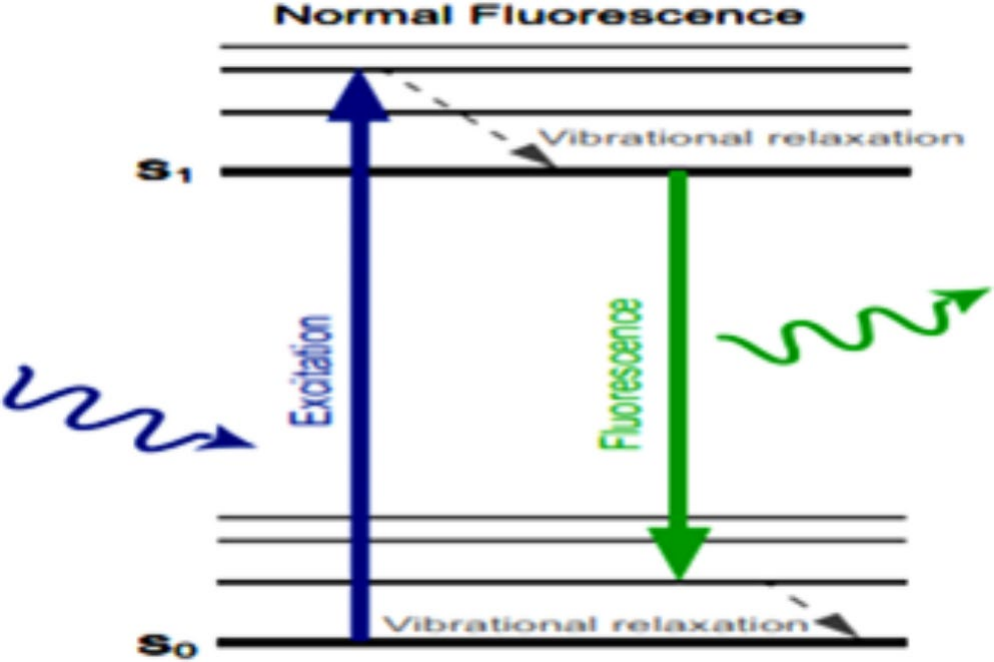
Jablonski diagrams. (**A**) Example of the electronic states of a fluorophore that is excited by a light with the appropriate wavelength (e.g., Blue). The transition from low-energy electronic state S_0_ to a higher vibrational ground state, S_1_, is caused by the absorption of energy given by the excitation light. The return to the S_0_ electronic state generates the emission of a photon (fluorescence) of lower energy (longer wavelength, such as green) [15]

The fluorophore material is a fluorescent chemical compound, which has re-emitting property of the light at the light excitation. Usually, it consists of several combined aromatic groups or planar or cyclic molecules with several π bonds [13]. A certain light wavelength is absorbed by a fluorophore material and is emitted at a longer wavelength. The wavelengths that are absorbed, the efficiency of the energy transfer, and the time before the emission process, all of them are dependent on both the fluorophore structure and its chemical environment, as the molecule in its excited state interacts with surrounding molecules [14]. Usually, the emission spectrum is sharper than the excitation spectrum, so, it has a longer wavelength and correspondingly lower energy. The excitation energies are in wavelength range the ultraviolet through the visible spectrum and emission energies extend from the visible light into the near-infrared region [15, 16]. As shown in Fig. 2.

The structure and chemical environment of the fluorophore is the main base that produces its property of absorption light energy at a specific wavelength and re-emitting it at a longer wavelength. Also, the absorbed wavelengths, the energy transfer efficiency, and time before emission depend on both.

### Quantum Dotsnanomaterial

In the 1980s, The QDs was discovered; it has been credited to Brus, Efros, and Ekimov by the Optical Society of America. Notably, they present an individual size and shape that produce unique optoelectronic properties, which have found useful applications in bio-imaging in recent years [17].QDs are nanocrystals collected of a core of semiconductor material, surrounded by a shell of another semiconductor, which has a larger spectral band-gap. Usually, QD cores are composed of elements from groups II and VI, i.e. CdSe (most common) or groups III and V, i.e., InP, and the shell material must have a high band-gap. An idealistic QD has a diameter in the range 2–10 nm, which makes it to a size that allows one-on-one interaction with biomolecules such as proteins. [18].

#### Properties of QDs

As a result of QD’s small size, it behaves differently from bulk solids. The fluorescent properties of QDs are engendered from the fact, that their excitation states/band gaps are spatially confined. The band-gap physical size sets the photon‘s emission wavelength, which can vary from UV to NIR wavelengths (400–1350 nm) [17].The adverse relationship between the nano-crystal size and energy band-gap is the most well-documented and understood property [18]. QDs are inorganic fluorophores that have size-tunable emission, light absorbance strongly [19], fluorescence brightly [20], symmetric emission bands narrowly [21], and high photo-stability [3].

The optical properties of QDs are preserved with superb stability at conjugation to biomolecules; in addition, it can be excited by a single light source only. Although white light (with its many different wavelengths whose many colors) can excite QDs. Finally, the QDs have a long fluorescent lifetime after excitation, which can be advantageous in time-gated imaging [22].

#### The Challengeof Using QDs

In contrast to organic fluorophores, QDs have some unique photo-physical properties [23]. The unique properties of QDs that; they have 20 times as bright and 100 times as stable against photo-bleaching when compared to conventional fluorophores [24, 25]. QDs is highly promising fluorescent tags for using in biological applications via significant superiority over classic organic fluorophores. Table 1 displays a comparison between QDs and organic fluorophores (dyes).

**Table 1:** Comparison between QDs and organic fluorophores

Optical factors	Quantum dots	Organic fluorophores	Reference
Excitation	A wide range of UV light excites a QD for any size.	Narrow excitation spectra	[28]
Emission bandwidth	20–40 nm	50–100 nm	[29]
Fluorescence lifetime	10–40 NS	Few nanoseconds	[30]
Photostability (upon constant illumination)	Stable for over 14 h	Fluorescein photobleaches completely in under 20 min	[31]

##### Types of QDs

There are three main types of quantum dots; the first type is (III-V-semiconductors), the second type is (II-VI- semiconductors), and the third type (Silicon) that is standard material of the semiconductor and chip industry [26]. It’s known, the ideal QDs consist of III-V, IV-VI, or II-IV semiconductor core (e.g., InAs, CdTe, CdSe, GaAs, PbSe, InP, and GaN [29].The core is covered by a wide band-gap semiconductor shell for minimizing the surface deficiency and enhancing the quantum yield [28–30]. One of the major hurdles, which hinder the application of III-V, II-IV, or IV-VI QDs in Vivo biological research, is the worry about the toxicity associated with the cadmium, lead, or arsenic- QDs using [31, 32]. Also, it has many features over common fluorescent organic dyes; resistance to photo-bleaching, wide emission range from VIS to the IR region with relatively high QYs [33]. In applications of Vivo, fundamentally Si QDs degrade to silicic acid that can be excreted through urine. In Vitro, the use of Si QDs is safer than Cd-containing quantum dots, as a common QD element, 10-times [32, 34].

##### QDs Toxicityand biocomatibility

It’s so important to deal with biocompatibility and toxicity of QDs for using it in biology. The surface of nano-materials is active, as a result of its large surface area and surface to volume ratio. Then, it is necessary to exclude the effect of solubility and possible contamination, which also would decrease the validity of any toxicity testing [35]. The QDs toxicity, as a function of physicochemical and environmental factors is paraded by Hardman in 2006 [36]. The fundamental factors that are responsible for the QDs toxicity value and biocompatibility are; size, charge, concentration, capping material bioactivity, functional groups, photolytic stability, and mechanical stability [37, 38].

## Challenge of QDs Nanomaterial Preparation

**Fig. 3:**
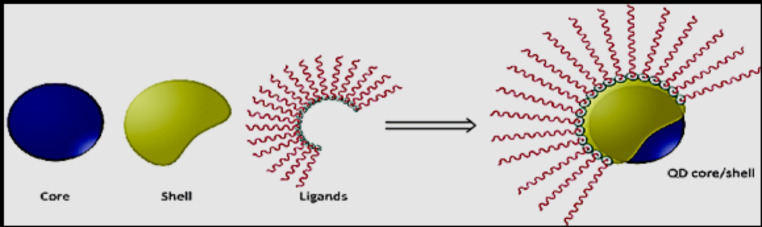
Basic architecture of core shell ligands in QDs [39]

The QDs preparation process is for gaining robustly luminescent nano-crystals that are stable, biocompatible, and soluble in aqueous solutions. Consequently, the core material (semiconductor) must be snug from degradation and oxidation to optimize QDs performance. Another problematic issue is the elevated reactivity of QDs cores that produce unspecific interactions with macromolecules, which leads to particle aggregation and fluorescence variation. The shell growth process and surface modification promote the stability and photoluminescence enhancement of the core, as shown in Fig. 3. The latest one, and probably most important is the QDs toxicity which can be greatly decreased using a surface modification as well [39].

### Synthesis of only core in Quantum dots

There are two general methods for the QDs preparation, which are displayed in the last decade: (1) the nano-size formation of semiconductor particles using colloidal chemistry [40], (2) the epitaxial growth and/or nano-scale patterning is used [41], i.e. Utilizing lithography-established technology [42]. Most of the classical biological applications of QDs involve Cd-based nanoparticles [43–45]. The size of QDs, respectively their optical properties, can be controlled by various factors, such as the synthesis temperature [46], the precursor molar ratio, and the composition of reaction medium (matrix).

### Synthesis of core-shell Quantum dots

A core-shell QD consists of a semiconductor nano-crystal core that’s coated with another semiconductor material as a shell. The core material semiconductor of the core-shell QDs that has a narrow band-gap (CdSe), on the other hand, the shell material semiconductor has a wide band-gap (ZnS or CdS) [47]. The core-shell nano-crystals typically have brighter fluorescence and are more stable against photo-degradation than the core-only QDs [48]. The fluorescence quantum yield of core-shell nano-crystals can be 50–80%, however a fluorescence quantum yield of up to 40% is usually achieved (i.e. (CdSe/ZnS)) QDs.

### Hydrophilization of Quantum dots

The assembly of the utmost extremely used colloidal semiconductor QDs that included metal Chalcogenides (i.e. Sulfides, Tellurides, and Selenides) [49]. It’s depends on the employment of organometallic precursors (Dimethyl cadmium), metallic oxide (CdO), or organic acids and metallic salts inorganic (Nitrate, acetate, stearate) [50]. Water-insoluble QDs can be grown in hydrophobic solvents easily, however, the solubility in water needs sophisticated surface chemistry alterations and presents a significant challenge [51]. There are three common strategies for solubility of QDs in water: 1st ; ligand exchange, 2nd ; micelle formation during hydrophobic interaction, and 3rd ; silica encapsulation [52].

### Solubilization of Hydrophobic QDs

#### Ligand Exchange (Non-aqueous QDs)

**Fig. 4:**
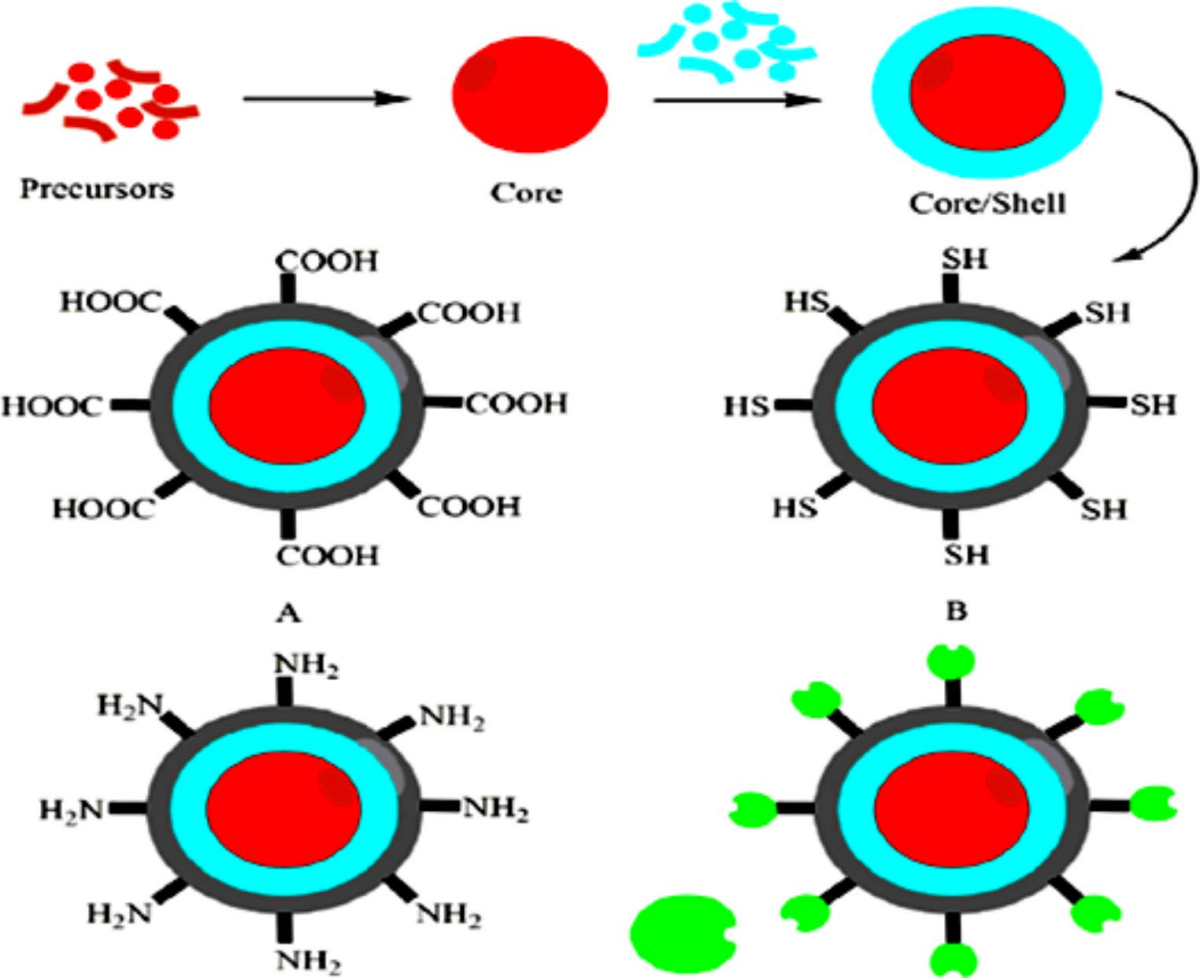
Chemical diagram of the bio-functionalized core/shell QDs. A, B, C andD are carboxyl, thiol, amino and streptavidin coated core/shell QDs, respectively [54]

The 1st technique embraces the ligand exchange process (at times called cap exchange). The native hydrophobic ligands are replaced by water-soluble bi-functional molecules in which one end connects to the QDs surface and the other end is hydrophilic and may also be reacting to Bio-molecules, as shown in Fig. 4. Nevertheless, this approach can negatively alter the chemical and physical states of the QDs surface and cause a dramatic decrease in quantum efficiency. And next, most of the water-soluble Bi-functional molecules are expensive and unstable [53].

#### Silica Encapsulation

The 2nd route is a native surface modification; adding of a silica shell to the nanoparticles by using a silica precursor during the poly-condensation [24]. Amorphous silica shells can be further functionalized with other molecules or polymers. Polymerized silica shells with polar groups using a silica precursor (functional organosilane molecules containing –NH_2_ or –SH, during the poly-condensation to insulate the hydrophobic QDs. Silica coating enhances the mechanical stability of colloidal QDs and protects them against oxidation and agglomeration. It covers a much broader pH range with comparison to carboxy-terminated ligands, which limit the QDs dispersion to basic pH [38].

#### Micelle Formation through Hydrophobic Interaction

The 3rd method maintains the native ligands on the QDs and uses various amphiphilic di-block and tri-block copolymers and phospholipids to tightly interleave the alkyl-phosphine ligands through hydrophobic interactions [54, 55]. In addition to the performance water solubility, the surface ligands act a critical function in insulating, passivation, and protecting the QD surface from decay in biological media. A more promising approach is to use long chain-length amphiphilic polymers to form micelle-like structures and hence to transfer the hydrophobic QDs into water [38].

### Preparation Techniques of QDs

#### Aqueous Synthesis of QDs

The most used way is aqueous synthesis, which is directly producing water-soluble QDs with premium biological compatibility and stability. QDs produced in an aqueous way display good reproducibility, low toxicity, and less cost. Essentially, the synthesis water-soluble QDs process occurs in a reflux condenser. Ordinarily, the reaction contains heavy metal (Zn, Cd, etc.) precursor with chalcogen precursors [38].

#### Microwave Irradiation Synthesis

The microwave technique is found to be the most effective method for preparing high-quality QDs in one-pot and in a shorter time [56]. Rapid homogeneous heating is the main base of the MW technique that realized through the microwaves penetration, it’s used to produce regular products with higher QY [57].

#### Micro-emulsion Synthesis

The micro-emulsion synthesis is highly reproducible, inexpensive and simple method enabling excellent control of QDs shape and size [58]. The water-to-surfactant molar ratio alteration is an important rule, which commands the QDs particle size. Nevertheless, the micro-emulsion synthesis produce slow yields of product; even large amounts of surfactant and organic solvent are used with comparison to bulk aqueous precipitation. The extraction of the nanoparticles from micro-emulsion is the key point of this procedure, into aqueous phase and to maintain their surface features and structural [38].

#### Lithography Synthesis

Lithography technique is known as a latter route for the QD preparation [59]. There are many disadvantages of the lithographic technique that are; the production of formation of defects, poor quality of the interface, contamination, non-uniformity size, and bit damage to the bulk of the crystal [60]. In addition, there is found that classic top-bottom modeling routes as photolithography and e-beam lithography, which are expensive and time-consuming processes. So, it’s a request for new QDs fabrication advanced techniques [24, 61–65]. The QDs toxicity problem can completely suppress by using non-toxic titanium dioxide in Vitro applications. That’s as a result of the enhanced photo-catalytic effect of TiO_2_ in the skin and other cancer types [66].

## Equipment of cell Analysis and Imaging

The most powerful and versatile technique is Fluorescence microscopy, which is used in the studying process of the biological molecules, aisles, and proceedings in living cells and tissues. The term “Super-Resolution Fluorescence Microscopy” marks various optical microscopy techniques, which have achieved optical resolutions over the diffraction limit, producing an improvement in spatial resolution [67, 68]. So, these are ideal for multi-color imaging applications [69].

The images of the biological sample are at the resolution value lower than 12 nm, so the NSOM is the SuperResolution Technique [70]. The lateral resolution (opening diameter) is fixed by ranges typically between 20 and 120 nm. However, NSOM has a dangerous disadvantage, that’s the extremely short distance between the opening and the irregular biological samples (1–10 nm), so it never is used for intracellular imaging. So, the QDs are successfully used as a cell marker used in NSOM as a result of exhibiting excellent intensity of fluorescence and perfect photo-stability.

The wide-field microscopy is one of the most important in super-resolution techniques, which can lower the diffraction limit the spatially modulates the transition between fluorescent and non-fluorescent states [71]. Temporal modulation is achieved via stochastic photo-switching/fluctuation and the localization analysis approach as used in photo-activated localization microscopy (PALM), fluorescence photo-activated localization microscopy (FPALM) and stochastic optical reconstruction microscopy (STORM).

STED uses spatially modulated and storable transitions between two fluorophore states – fluorescent and non-fluorescent – to break the diffraction limit by utilizing the non-linear depletion of fluorophores, selectively inhibiting fluorophores surrounding the center of an exciting spot by stimulated emission that contains two coincided laser beams [72]. It can emit fluorescence, generating a focal spot with a size equal to the center of the opening, thereby increasing the lateral resolution down to 20 nm.

## Branches of Bio-imaging Applications

Previously, different biological tags were utilized in different bio-imaging techniques. Discovery of new tags leads to fostering a completely new set of biological tagging methods. For example, discovering fluorescence microscopy (FM) in 1994, after the discovery of an alternative to traditional fluorescent dyes [82]. Noticeability, QDs has very suitable sizes for cell labeling, which has unique optical and electrical properties [83], systems are established for surface functionalization and multimeric binding capacities [73]. Thus, QDs reinforced targeting efficiencies, for long-term PL stability areas, high brightness, and multi-color detection [74, 75].

## Application of QDs in cell Analysis and Imaging

The scientific rampage was built on the chemical interactions recording between biomolecules with each other, also with different ionic and molecular species in the bio-imaging technique that takes place in cells and tissues [73]. The commonly utilized size of QDs ranges from 2 to 20 nm in diameter, which based on the precise structure of the material. Concurrently, QDs’ cores that include 200–10000 atoms have elevated molar extinction coefficients [74], and its electron density is sufficient, which provides high disparity in bio-imaging. As previously discussed, QDs have a specified surface functionalization. The surface layer is covered by ligands in producing biocompatible property, soluble ability, and good stability of QD in the bio-matrix. Then the QDs are conjugated with bio-detection elements for achieving the specific or non-specific targeting [75].So, the final QDs size allows them perfectly for internalization by cells different pathways. [73].

### Application of QDs Bio-imaging

#### In Vitro and in Vivo Imaging with QDs

Due to these distinctive optical properties, QDs have been used successfully in a variety of imaging applications for both in Vitro and in Vivo diagnostics [76, 77]. QDs have been successfully utilized as fluorescent tags for a variety of bio-analytical purposes, including the detection of DNA, proteins, and other biomolecules, labeling of cells, fluorescence resonant energy transfer (FRET)-based binding assays [78, 79], and the detection of other biomolecules [80, 81].

Kang et al. used QD-conjugated aptamers to have a look at the expression of different cancer markers concurrently inside the equal cancer cellular. They conjugated three extraordinary QDs (with three awesome emission wavelengths: 605, 655, and 705 nm) the usage of three aptamers (AS1411, TTA1, MUC-1, focused on specific cancers), respectively, after which discovered the multiplex imaging image. The fluorescence photographs for numerous cells were acquired and analyzed, and the triple cellular imaging of 3 specific QDs turned into effectively performed. QD-aptamers with high fluorescence intensity confirmed comparatively true fluorescence signals at the mobile surfaces [82]. To obtain qualified profiles, QDs are vital for cell imaging. Non-blinking QDs consisting of person lanthanide-doped up-converting nanoparticles [83] and zinc blended CdSe/CdS center/shell QDs [84] had been said, which solved the long-standing on/off emission hassle and provided strong upconverted luminescence whilst applied in mobile imaging [85].

Cao et al. passivated C-dots by means of poly (propionylethyleneimineco- ethyleneimine); ethyleneimine fraction, 20%. The treated C-dots have become with no trouble soluble in water, and have been additionally located to be strongly emissive within the visible wavelength range when excited by the usage of a 458 nm argon ion laser and an 800 nm femtosecond pulsed laser. Experiments confirmed that the C-dots could efficiently label both the cellular membrane and cytoplasm of MCF-7 cells, but did not attain the nucleus. C-dots exhibited -photon luminescence photographs at 800 nm excitation, showing fairly promising ability for cellular imaging [86]. Zrazhevskiy et al. advanced an advanced approach named multicolor multi-cycle molecular profiling (M3P). The M3P generation can create tricky quantitative molecular profiles in parallel for residing cells. They designed an established platform based totally on QD–protein A (named QD–SpA), and used the platform to put together a library of purposeful QD–antibody (called QD– SpA–Ab) probes quick and flexibly [87].

#### Early Diagnosis of Cancer by Quantum Dots

**Fig. 5:**
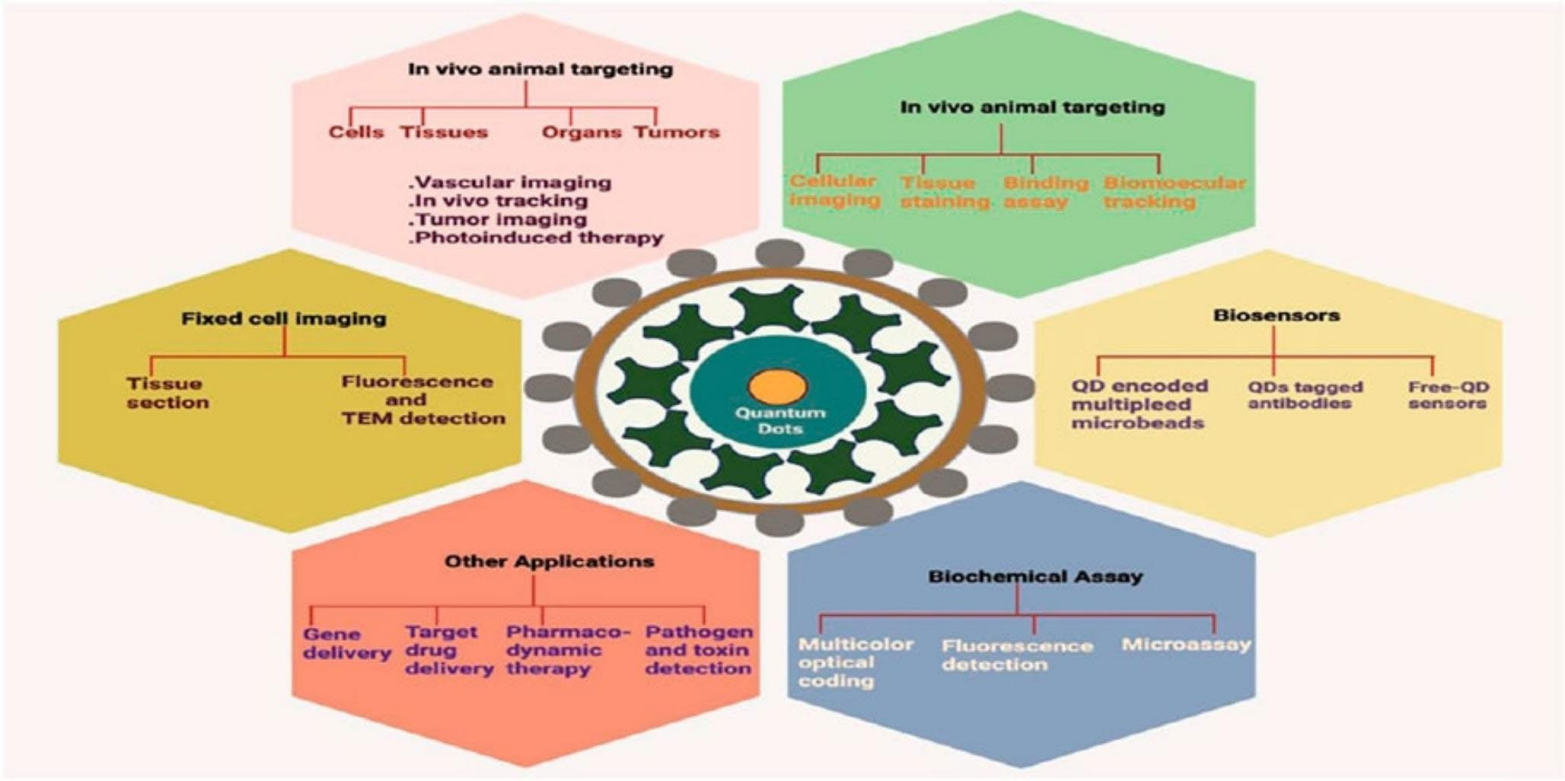
Various applications of Quantum dots in bioimaging and diagnosis

QDs can serve a vital element in the analysis of tumors. The detection of phase I disorders is related to a 5-years staying power rate of more than 90% in the sizable majority of instances due to remedial therapy. Fluorescence naming or fluorescence imaging technologies are crucial equipment in the medical decision armory. Due to their photochemical stability and sturdy fluorescence output, quantum dots are mainly used to improve fluorescence imaging Fig. 5 [88]. QDs are broadly employed in biomedical programs which includes reading delivery mechanisms in cells, cellular purposeful heterogeneity, diffusion actions of membrane shipping proteins, nonspecific labeling for imaging and detection, the assessment of blood and lymph vessels (which include micro-vessels), prognosis of hepatoma in Vivo, intracellular shipping, internalization of QDs by means of stay cells, probes in different bioassays, and many more [89]. Chung et al. used microwave irradiation to create fluorescent iron oxide and carbon dots-based totally nanoparticles for cancer mobile fluorescence imaging and treatment. Using these QDs as fluorescent probes in most cancers mobile traces revealed that they were much less risky and allowed for brief and quantitative imaging [90].

#### In-Vitro Tumor Imaging

Numerous researches used Quantum dots to broaden in Vitro fluorescent photos of human malignant cells from cancer, ovarian, breast, pancreatic, glioblastoma, ovarian epidermoid, lung, hepatocellular, and adenocarcinoma cancers. According to Zhang et al., anti-type-1 insulin-like increase component receptor (IGF1R) quantum dots are a possible alternative for focusing and imaging breast most cancers cells. The invention of up-regulated IGF1R in MCF-7 breast most cancers cells using Quantum dots-antagonistic to IGFR1 shape changed into essential in that studies. The epidermal increase element receptor (EGFR), additionally known as human epidermal receptor aspect 2 (HER2), is a trans-membrane gp that belongs to the erbB circle of relatives of tyrosine kinase receptors. It can bind to epidermal growth thing (EGF). The Quantum dots-EGF form can be applied for diverse malignancy cells fluorescence imaging considering the fact that EGFR is over-communicated in lots of sicknesses [91, 92]. For controlling apoptosis and autophagy in B16F10 cancer malignant cells and in Vitro imaging, Bajpai et al., employed an unmarried-step warmness remedy to synthesize nitrogen-phosphorous doped carbon dots. Researchers assessed various parameters like cellular viability, morphology, fluorescent stay-dead mobile check, mitochondrial capability assay, siRNA transfection mobile bio-imaging, and many others to validate its anticancer capability [93].

One of the most sizable methods for in Vivo focusing and imaging is to study the bio-distribution and pharmacology of the useful Quantum dots. The type and composition of natural or bioorganic shells of Quantum dots decide their biocompatibility. Tumor biomarkers are critical in detecting and diagnosing cancer at an early stage. If a number of tumor markers may be determined and correctly differentiated among carcinogenic and non-carcinogenic cells, biomarker checking out is probably useful for cancer screening and analysis [94]. Angiogenesis is required for the increase and mobility of tumor cells. Integrin v3, which binds to RGD-included interstitial lattice segments, suggests a crucial function in tumor angiogenesis and metastasis. Quantum dots provide an exceptional device for tumor vascular imaging and multimodal atomic imaging of angiogenesis. Quantum dots are new illumination nanoparticles with particular visual features and well-off plane technology, making them perfect as representation exams or transporters for distinguished conveyance and treatment programs. accordingly, practical Quantum dots which can execute imaging and drug conveyance obligations without the utilization of outside colours have been produced [95]. Yakavets et al., used ternary copper indium selenide/zinc sulfide-based QDs emitting inside the close to-infrared (750 nm) coupled to A20FMDV2 (peptide) to goal v6 integrin-rich head and neck squamous malignant cells. Nano-probes for imaging-guided surgical operation and NIR bio-imaging can be made with produced QDs [96]. Due to a high demand in biomedical imaging, Kwon et al., designed iron selenide (FeSe) QDs for in Vivo multi-photon imaging [97].

#### The Use of QDs in Medical Microbiology

Immune-fluorescent antibodies (IFA) are frequently used in ELISA-style detection procedures for bacteria and other microorganisms [98]. Given their excellent optical properties, it is not surprising that QDs have also been researched as a potential fluorophore to replace traditional natural dyes for such packages [99]. In order to connect 565 and 605 nm emitting CdSe/ZnS QDs to specific antibodies for Giardia lamblia and Cryptosporidium parvum, respectively, Zhu et colleagues used the non-covalent Avidin-biotin interaction [100]. Comparing the performance of a biotinylated antibody specific for the pathogenic bacterium Escherichia coli serotype 0157:H against that of a commercially available fluorescein iso-thio-cyanate (FITC) E. coli detection kit, Hahn and co-workers [101] used a similar methodology. Zhao et al. demonstrated multiplex detection of the three microorganisms in a complex mixture [102] using a combination of magnetic microparticles with three capture antibodies attached (specifically for S. typhimurium, S. flexneri, and E. coli O157:H7) and three exceptional sized CdTe QDs (with emission maxima at 520, 560, and 620 nm).

The QD reporter conjugates were added after a magnet was used to return the micro-particle-capture antibody to the bottom of a micro-centrifuge tube. Undoubtedly, when each of the target bacterial strains increased in number from 103 to 106 CFU, it was seen that the depth of the alerts at 525, 570, and 625 nm increased linearly. Due to the diverse excitation wavelengths necessary for three distinct dyes today, as well as the potential for overlap in their typically wider emission spectra, such multiplexing functionality could be difficult, if not impossible, to achieve using conventional dyes. Edgar et al. coupled CdSe/ZnS QDs to E. coli-specific T7 bacteriophage particles as an alternative to the usage of antibodies [103].

#### QDs for Labeling Cells

The optical homes of QDs, mainly the wavelength in their fluorescence, rely strongly on their length. Because of their reduced tendency to photo bleach, colloidal QDs are interesting fluorescence probes for all sorts of labeling studies. As QDs have steady and unique optical properties, they are utilized in cellular marking [104, 105]. QDs can concurrently tag multiple inter and intracellular additives of stay cells for time durations ranging from seconds to months. Distinctive colorings of QDs can label variant mobile components that can be without difficulty visualized with fluorescent microscopy or in vivo [106]. for instance, plant bioimaging: CdSe QDs bind generally to cellulose and lignin in the cellular wall and consequently provide a fluorescent picture of plant cells; animal bioimaging: biotinylated Cholera toxin B (CTxB) with QD–avidin conjugates for labeling of ganglion [107]; CHPNH2 QD nano-gel has potential for use in long-term cell imaging; prokaryotic bioimaging: for measuring the bacterial mobile, middle magnetic beads which can be anti E. coli 0157-lined and streptidine-coated are used [108].

#### Identification of Molecular Fingerprints of Diseases

Molecular fingerprinting of illnesses implies characterization of biomarker expression schemes in diseased cells in assessment to healthy ones. QD-based probes are uniquely suited for this project when hired by both multi-parameter flowcytometry analysis of mobile populations and quantitative multiplexed analysis of biomarker expression in intact tissue specimens. For instance, Chattopadhyay et al., by using utilizing a 17-parameter go with the flowcytometry (based on eight QD probes and nine natural fluorophores), discovered huge phenotypic differences among T-cells particular to awesome epitopes of the same pathogen [109]. Get entry to molecular profiles of character cell populations not handiest improves our understanding of immune reaction, but also allows evaluation of modifications taking place all through immune system problems, touchy detection of metastasizing cancer cells in a bloodstream, and correct phenotyping of heterogeneous mobile populations. Transferring in the direction of introducing QD era into scientific diagnostics, five-parameter characterization of breast most cancers tissue specimens received from biopsies has been tested [110].

## Therapeutic Application

So, in the recent advances of the QDs surface modification, it has enabled in cancer imaging using their application. Also, the QDs can conjugate with biomolecules, i.e. Peptides and antibodies that may be targeted tumors in Vivo.

### QD Based Theranostics

We begin this section by giving examples of how QDs and cytotoxic medicines can be coupled to create ensembles that have both therapeutic and diagnostic value. The main characteristics of QDs that make them desirable for use in such constructs are: (i) their nanoparticle size means they exhibit the aforementioned EPR effect; (ii) their high single and two-photon extinction co-efficient means they can be used to image deeply within mammalian tissue; and (iii) their broad absorption and size-tunable emission spectra offers a wide selection of potential excitation and emission wavelengths for improved imaging. The first instance we address concerns a study by Bagalkot et al., who used carbodimide-based chemistry to covalently bind an amine ended prostate specific RNA aptamer (A10 PSMA) to carboxylic acid functionalised 490 nm emitting CdSe/ZnS QDs [111]. Wang et al. have created a pH sensitive CdSe/ZnS - Dox conjugate for pH mediated release of Dox using a poly(methacrylate) comb type co-polymer with both amine and PEG terminated side chains as a capping ligand [112]. This strategy is justified by the notion that healthy tissue typically has a pH higher than that of malignant tissue. Surprisingly, the authors exploited the multidentate amine side chains as a multidentate ligand for attachment of the polymer to the QD surface and subsequently linked Dox to the polymer coated QDs via a pH sensitive imine bond (compared to greater affinity thiol groups).

Zheng et al. employed carbon quantum dots (CQDs) as an alternative to CdSe/ZnS QDs as the fluorescent reporter in an effort to get around the toxicity concerns associated with Cd-based nanoparticles for in vivo applications [113]. CQDs, a more recent addition to the QD family, can be made from easily available, reasonably priced carbon precursors such graphite, glucose, or citric acid [114]. CQDs exhibit excitation dependent emission spectra, in contrast to their semiconductor counterparts, where the emission maxima red-shifts with increasing excitation wavelength [115]. These nanoparticles have strong two-photon absorptions, good fluorescence quantum yields, and can be coated with biocompatible ligands to make them appropriate for biological applications, however the cause of this phenomena is still not entirely understood [116]. Citric acid passivated with an amine ended polymer was how Zheng et al. created CQDs [117]. Fakhroueian and coworkers reported the use of Zinc Oxide QDs as standalone chemotherapeutic agents as an alternative to using QDs in conjunction with chemo drugs [118]. This study demonstrated an enhanced cytotoxic impact in cancerous cell lines compared to the non-cancerous cell line examined after incubating both cancerous and non-cancerous cell lines with oleic acid coated ZnO QDs. These QDs displayed anti-fungal and anti-bacterial characteristics in addition to their anti-cancer actions.

### QDs for Use in Photodynamic Therapy

**Fig. 6:**
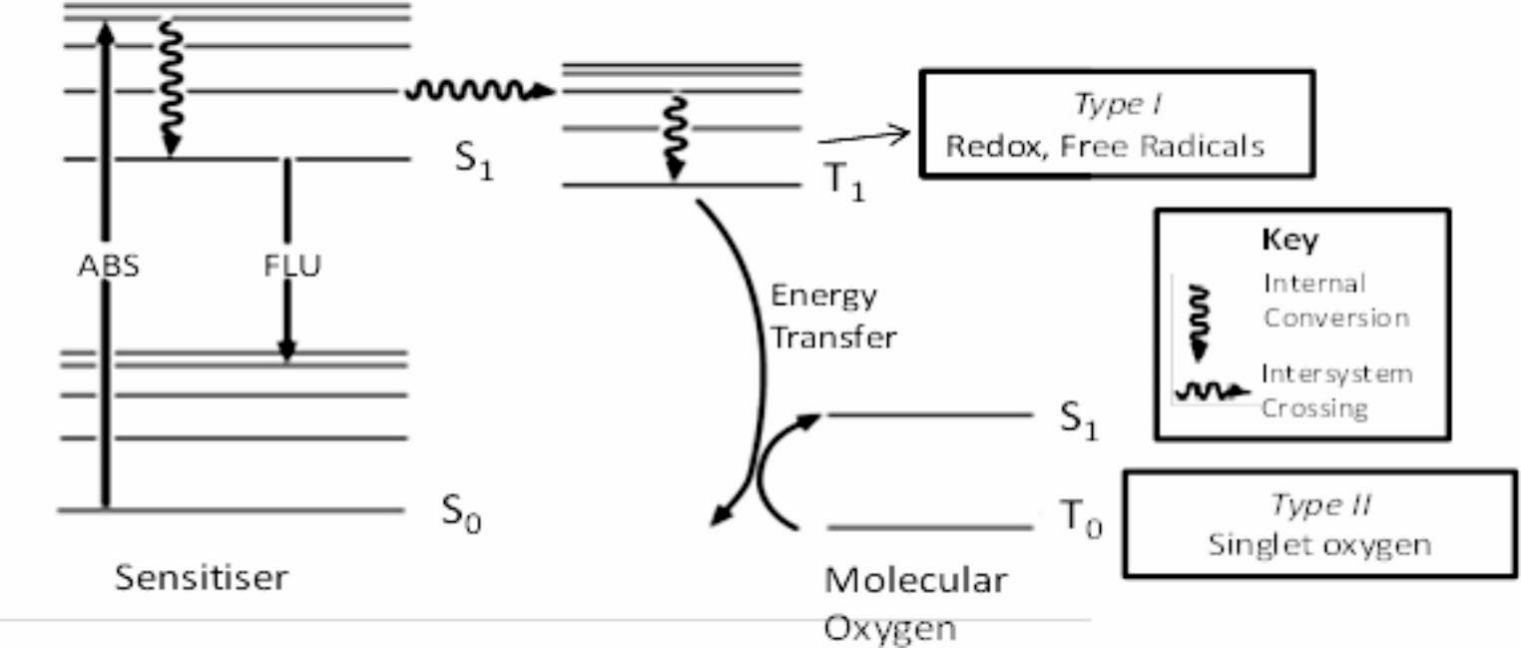
Modified Jablonski Diagram illustrating the key photophysical processes involved PDT

A medical procedure known as photodynamic therapy (PDT) kills sick cells by utilising a mix of light, photosensitizing medications, and molecular oxygen. The photosensitizer (PS) molecule typically uses a light source to excite an electron from the ground state (S0) to the first excited singlet state (S1), as seen in Fig. 6.

The excited electron has a few alternatives at this point. It has two options: either it returns to the ground state and releases its excited energy as fluorescence (FLU), or it engages in intersystem crossing, which causes the excited electron’s spin to flip, creating a triplet excited state (T1). Triplet states frequently result in phosphorescent emission and have a substantially longer life span than singlet states. However, triplet excited states can also participate in energy or electron transfer with molecules like water or proteins to create free radicals that are known as reactive oxygen species (ROS). Strong intracellular cytotoxins known as ROS eventually cause cell death [119].

The use of PDT as a therapeutic therapy has dramatically increased since 1993, when the first PDT agent (Photofrin®) was authorised for use in clinical settings. PDT is increasingly being examined as a treatment for different malignancies, including head and neck, lung, oesophageal, prostate, and bladder, despite being historically thought of as a treatment for skin cancers. Additionally, it can be used to treat some non-cancerous disorders, like psoriasis [120]. However, a number of issues have prevented PDT from being used more frequently in clinics. For instance, the visible region (below 700 nm) absorbs in the currently approved photosensitsers, limiting light penetration to only a few mm. For surface tumours, this is the right course of action, but it is inappropriate for deeper-seated tumours. The light intended for PS activation may not reach the target molecules in situ because endogenous substances, including melanin or heame pigments, compete with PS for light absorption [121].

If PS are produced that absorb in the near infrared (NIR) region, where PS absorbance is significantly greater, this interference can be reduced. In fact, the depth of light penetration through skin is four times greater at 800 nm (8 mm) than at 630 nm (2 mm) [122]. Additionally, the PS should preferably not be hazardous in ambient light and have no dark cytotoxicity. To date, patients need to spend many weeks under low level lighting after receiving treatment with first generation photosensitizers in order to prevent significant skin burning. In order to prevent harm to healthy cells, the PS should be as selective as possible for the targeted tissues. Otherwise, healthy tissue is exposed to radiation due to a lack of selectivity and poor visualisation of the afflicted tissue, which increases the likelihood of adverse effects [123]. Choi et al. created a CQD made from a precursor of -cyclodextrin passivated with poly(ethyleneglycol) diamine covalently linked to folic acid in an effort to optimise the targeting of PS medicines for use in PDT [124].

It must be noted that these outcomes are for a single treatment, and as is typical with conventional chemotherapy, one would anticipate greater benefits following subsequent treatment cycles. While the prior instances largely used QDs as fluorescent tracers, our lab and others are also looking at the possibility of using QDs in two-photon excitation PDT (TPE-PDT) [125, 126]. Two-photon excitation is being looked into as a potential excitation source in PDT due to its enhanced capacity for tissue penetration and spatial resolution [127]. In fact, two-photon excitation has been used to successfully activate sensitizers to a depth of 2 cm in breast and lung cancer Xenografts [128]. However, currently approved sensitizers, like Photofrin, require extremely high two-photon excitation levels that are near to the threshold of tissue damage because they cannot efficiently absorb two-photon irradiation. In fact, Karotki et al. hypothesised that in order for sensitizers to be therapeutically useful, they would need two-photon absorption levels that were two to three orders of magnitude higher than those of Photofrin [129]. Contrarily, QDs with two-photon absorption cross sections as high as 48 000 Göppert-Mayer units can be easily stimulated by lasers with a power of just 1 mW [130]. Ge et al. reported using graphene QDs (GQDs) as “stand-alone” sensitizers with extraordinarily high 1O2 quantum yields (higher than unity) and broad absorption spectra that extended into the red [131], which is a very promising development. Minimal dark toxicity was seen across the whole concentration range when HeLa cells were treated with GQDs at escalating doses (0.036 to 18 M).

### QD: Unique Carrier for Drug Delivery

**Fig. 7:**
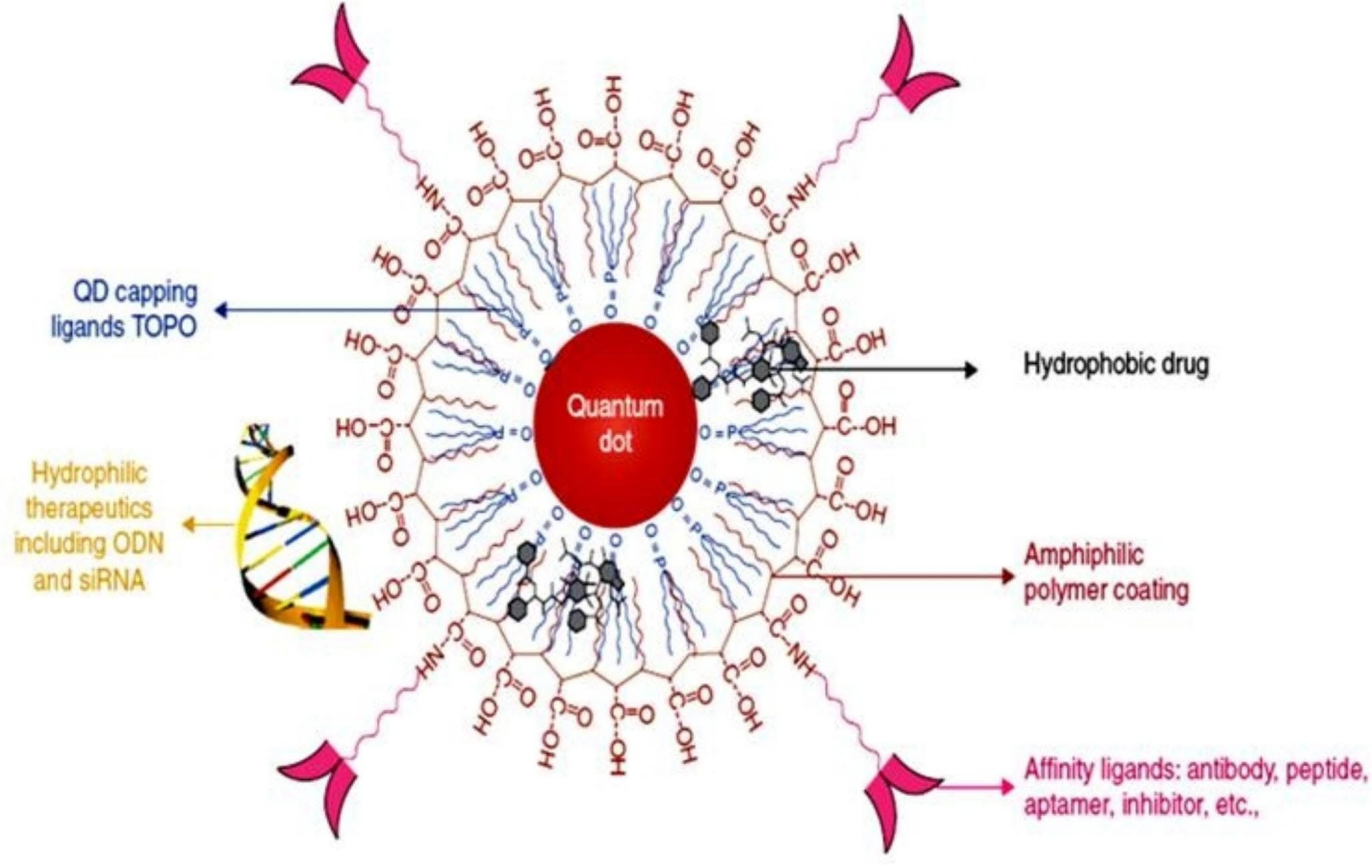
QD-based drug carriers integrate drug delivery tracing, loading of various types of drugs (e.g. hydrophobic small-molecule drugs between the QD core and polymer coating or hydrophilic drugs on the exterior surface of the polymeric shell), and targeting functionality. Intermediate size of such carriers ensures slower renal filtration as well as RES uptake, thus increasing blood circulation time and improving delivery efficiency [136]

Correct popularity of key molecular goals distinguishing not-healthy from healthy cells enables focused drug shipping with negligible side effects. Nanoparticle drug vendors reveal essential features for efficient focused delivery which include efficiently lengthy blood flow, protection the shipment from degradation, big drug loading capability, managed drug launch profile and integration a couple of targeting ligands on their surface. Moreover, QD probes have enough money novel capability of traceable drug delivery, as bio-distribution of vendors and intracellular uptake can be checked through fluorescence. Many drug transport applications the use of QDs was installed recently, as an instance, Chen et al. co-transfected QDs and siRNA the use of Lipofectamine 2000 and monitored transfection performance thru QD fluorescence. Blending QDs with transfection reagent in 1:1 mass ratio advanced dating between the QD sign intensity and the diploma of gene silencing [132]. Fascinatingly, more co-transfection of different siRNA molecules with various QD shades might expand multiplexed monitoring of gene silencing. More accurate quantitative data approximately the variety of siRNA molecules introduced into cells can be attained by means of the usage of QDdoped chitosan nano-beadsdemonstrated by Tan et al. [133]. On this method siRNA molecules are positioned downed at the floor of nano-beads, and intracellular shipping can be straightly monitored via the nano-beads fluorescence. Further improvement can be received using carbon nanotubes for intracellular delivery of antisense oligonucleotides tagged with QDs via Jia et al. [134]. As an instance, directly tagging of plasmid DNA with QDs followed through Lipofectamine-mediated transfection enabled long-term survey of intracellular and intra-nuclear localization and delivery of plasmid DNA, at the same time as keeping the potential of expressing reporter protein encoded via the plasmid85. Development of unmarried-QD drug shipping motors for in Vivo usage is admirable, as intermediate length of such providers (~ 10–20 nm in diameter) diminishes the renal clearance as well as uptake by Reticulo-Endothelial Machine(RES), accordingly growing the blood flow time and improving the shipping, as shown in Fig. 7.

### Targeted Gene Delivery

**Fig. 8:**
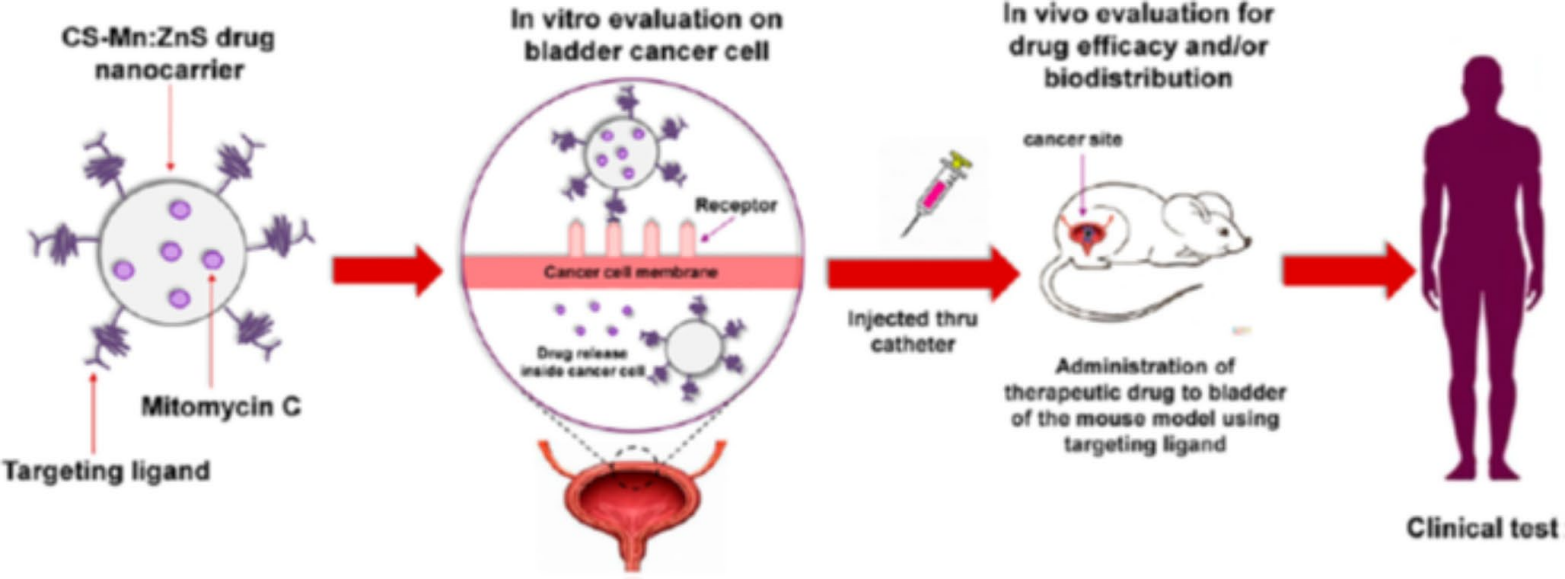
Schematic representation of mitomycin C encapsulated quantum dots–chitosan nanocarrier system for treatment of non-muscle invasive bladder cancer [136]

Quantum dots have also been shown to have a capability for delivering attributes extra in range, complexity, or each, such as small interfering RNA, similarly to small atom medicines (siRNA). The therapeutic si-RNA, which is small and two-fold abandoned, acts by means of preventing the outflow of undesirable, infection-causing residences. In line with Li et al., QDs could efficiently delivery siRNA into HeLa cells at the same time as retaining goal high-quality, and nanocomposites stacked with QDs could be used as fluorescence materials, taking into account monitoring and drawback of QDs amid deliverance and transfection [135]. Cas9 and gRNA have been transferred into CFT073, a UPEC strain, the usage of CQDs by way of Gupta et al. To make nanocomplex CRISPR-dots, the CQDs were chemically coupled to Cas9 and papG-focused guide RNA (gRNA). Focused on the virulence factor Fimbrial Adhesion (papG gene) and a bacterial adhesion molecule are their main potential [132]. Huang et al., designed CKAP4 antibody-conjugated Si quantum dot micelles for centered imaging of lung cancer. As shown in Fig. 8, in vivo fluorescence-imaging research discovered that the Si QDs micelles-CKAP4 were metabolized by way of the liver and removed via the kidney. Moreover, they exactly centered lung cancer tissue in vivo as compared with wholesome lung tissue [136].

## Conclusions

As a result of the spread of epidemics and viruses (for example; Covid-19) that affect the entire world, it was only natural, but it was necessary for the whole world to go to find diagnostic methods to help mankind face these health threats to all living things (plants or animals, of course, humans also). It’s given the importance of bio-imaging applications in the process of internal diagnosis of cells and tissues. And, as a result of using the organic compounds in the whole world with the apparent fluorescence emission. Despite its damage and its toxicity percentage to internationally recognized living cells, it was and still is used in many countries. So the eyes of the scholars turned to quantum dot nano-materials, which have the same property of the organic one, but with a higher safety rate than that. Hence the challenge in the preparation process for these materials.

The different types of quantum dot nano-materials are promising in the bio-imaging application field for their properties; long fluorescent lifetime after excitation, which can be advantageous in time-gated imaging, high brightness emission, low toxicity, and suitable bio-conjunction. In addition, it has several simple, safe, and inexpensive preparation methods. This allows it to be used in many important biological applications.The three main types of quantum dots; (III-V) - semiconductors, (II-VI) - semiconductors, and Silicon (Si), are already used in vitro and vivo imaging applications. But the first type is still the most used in many applications so far. And, that is a result of the possibility of its preparation using several ways, in addition to, the ease of size controlling that produces a wide range of fluorescence emission.

The most valuable and promising prospects in the fields of medication delivery, targeting, and imaging in recent years have been QDs. They are great prospects for in vivo bioimaging, gene/drug administration, and cancer detection due to their low toxicity, low cost, and strong biocompatibility. This has had a significant impact on a number of disciplines, including biotechnology, bioassays, intracellular tagging as photo sensitizers for cancer treatment, and illness detection.

In conclusion, because QDs have special size-dependent optical features, their use in cancer research has drastically expanded. Molecular imaging methods for in vivo research that are highly sensitive include bioconjugated near-infrared QDs probes. The application of QDs in the detection and localization of metastasis, the quantitative measurement of molecular targets to facilitate targeted therapy, the tracking of drug administration, and the noninvasive real-time monitoring of therapeutic efficacy are all possible with further research. According to recommendations for future work. QDs have considerable promise in imaging, clinical applications, and fundamental biomedical research. An examination of QD characteristics and popular perception is done in order to fully grasp the potential of these new generation materials. One major obstacle is the toxicity of these nanoparticles, which is still not well understood. We need to further investigate QDs’ possible toxicity before commercialising them for use in humans, which will take some time. The integration of materials or methods, such as magnetic and electric materials or signal application methods, is anticipated to result in powerful biosensing QDs in the future. Now, scientists will carry out the synthesis of QDs using an environmentally friendly method that calls for improved stability, biocompatibility, and distinctive optical features.

## Data Availability

Not applicable.
